# 4-Methyl-*N*-(3-oxo-2,3-dihydro-1,2-benzisothia­zol-2-yl)benzene­sulfonamide

**DOI:** 10.1107/S1600536809003201

**Published:** 2009-01-31

**Authors:** Corrado Rizzoli, Paola Vicini, Matteo Incerti

**Affiliations:** a Dipartimento di Chimica Generale ed Inorganica, Chimica Analitica, Chimica Fisica, Viale G. P. Usberti 17/A, Universitá di Parma, I-43100 Parma, Italy; b Dipartimento Farmaceutico, Viale G. P. Usberti 27/A, Universitá di Parma, I-43100 Parma, Italy

## Abstract

In the title mol­ecule, C_14_H_12_N_2_O_3_S_2_, the benzisothia­zolone ring system is essentially planar and forms a dihedral angle of 67.37 (6)° with the plane of the benzene ring. In the crystal structure, mol­ecules are linked *via* inter­molecular N—H⋯O and C—H⋯O hydrogen bonds to form chains parallel to the *b* axis.

## Related literature

For the chemical and biological properties of 1,2-benzisothia­zol-3(2*H*)-one derivatives, see: Clerici *et al.* (2007 ▶); Siegemund *et al.* (2002 ▶). For 2-amino-1,2-benzisothia­zol-3(2*H*)-one derivatives with anti­platelet/spasmolitic effects, see: Vicini *et al.* (1997 ▶,2000 ▶). For derivatives with anti­microbial properties, see: Vicini *et al.* (2002 ▶); Zani *et al.* (2004 ▶). For the synthesis of the title compound, see: Vicini *et al.* (2009 ▶). For the crystal structures of related compounds, see: Cavalca *et al.* (1970 ▶); Ranganathan *et al.* (2002 ▶); Steinfeld & Kersting (2006 ▶); Kim *et al.* (1996 ▶); Xu *et al.* (2006 ▶); Sarma & Mugesh (2007 ▶); Kolberg *et al.* (1999 ▶).
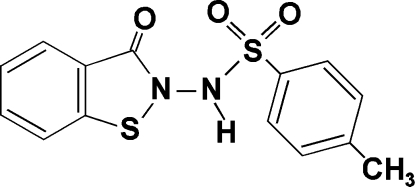


## Experimental

### 

#### Crystal data

C_14_H_12_N_2_O_3_S_2_
*M* *_r_* = 320.38
Monoclinic, 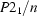

*a* = 8.051 (3) Å
*b* = 7.655 (3) Å
*c* = 23.910 (10) Å
β = 98.490 (8)°
*V* = 1457.4 (10) Å^3^
*Z* = 4
Mo *K*α radiation
μ = 0.38 mm^−1^
*T* = 296 (2) K
0.28 × 0.26 × 0.12 mm

#### Data collection

Bruker SMART 1000 CCD area-detector diffractometer
Absorption correction: multi-scan (*SADABS*; Bruker, 1997 ▶) *T*_min_ = 0.892, *T*_max_ = 0.957
17685 measured reflections
3521 independent reflections
1888 reflections with *I* > 2σ(*I*)
*R*_int_ = 0.041

#### Refinement

*R*[*F* ^2^ > 2σ(*F* ^2^)] = 0.040
*wR*(*F* ^2^) = 0.089
*S* = 1.01
3521 reflections
194 parameters
1 restraint
H atoms treated by a mixture of independent and constrained refinement
Δρ_max_ = 0.23 e Å^−3^
Δρ_min_ = −0.28 e Å^−3^

### 

Data collection: *SMART* (Bruker, 1997 ▶); cell refinement: *SAINT* (Bruker, 1997 ▶); data reduction: *SAINT*; program(s) used to solve structure: *SIR97* (Altomare *et al.*, 1999 ▶); program(s) used to refine structure: *SHELXL97* (Sheldrick, 2008 ▶); molecular graphics: *ORTEP-3 for Windows* (Farrugia, 1997 ▶) and *SCHAKAL* (Keller, 1997 ▶); software used to prepare material for publication: *SHELXL97* and *PARST95* (Nardelli, 1995 ▶).

## Supplementary Material

Crystal structure: contains datablocks global, I. DOI: 10.1107/S1600536809003201/lh2764sup1.cif

Structure factors: contains datablocks I. DOI: 10.1107/S1600536809003201/lh2764Isup2.hkl

Additional supplementary materials:  crystallographic information; 3D view; checkCIF report

## Figures and Tables

**Table 1:** Hydrogen-bond geometry (Å, °)

*D*—H⋯*A*	*D*—H	H⋯*A*	*D*⋯*A*	*D*—H⋯*A*
N2—H2⋯O1^i^	0.848 (17)	1.948 (17)	2.784 (3)	168.3 (15)
C6—H6⋯O2^ii^	0.93	2.56	3.492 (3)	175

Symmetry codes: (i) 
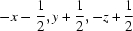
; (ii) 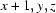.

**Fig. 1.**
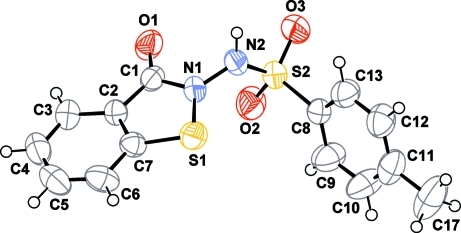
The molecular structure of the title compound, with displacement ellipsoids drawn at the 50% probability level.

**Fig. 2.**
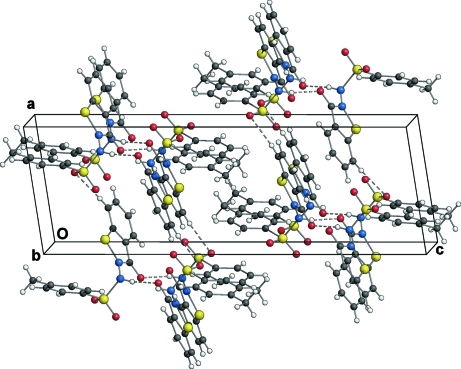
Crystal packing of the title compound viewed approximately along the b axis. Intemolecular N—H···O and C–H···O hydrogen bonds are shown as dashed lines.

## supplementary crystallographic information

### Comment

Over the past decades a substantial number of 1,2-benzisothiazol-3(2*H*)-one derivatives have been reported to possess a wide range of biological activities including antimicrobial, antiviral, anticancer, anti-inflammatory, cartilage antidegenerative and other pharmacological activities (Clerici *et al.*, 2007; Siegemund *et al.*, 2002). As part of our program aimed at developing novel biologically active 1,2-benzisothiazol-3(2*H*)-ones, we have synthesized in the last years 2-amino-1,2-benzisothiazol-3(2*H*)-one derivatives resulted in the discovery of new compounds active as antiplatelet/spasmolitic agents (Vicini *et al.*, 1997; Vicini *et al.*, 2000) and of compounds endowed with very interesting antimicrobial properties (Vicini *et al.*, 2002; Zani *et al.*, 2004). Recently, in our continuing efforts to find novel effective 2-amino-1,2-benzisothiazol-3(2*H*)-one derivatives, we have synthesized a series of 2-(phenylsulfonyl)amino-1,2-benzisothiazol-3(2*H*)-ones which exhibit anti-HIV-1 activity against wild type virus and against viral strains carrying clinically relevant mutations (Vicini *et al.*, 2009). Experimental evidences suggest non classical targets for this novel class of anti-HIV-1 agents. In order to study their binding sites at a molecular level we thought it appropriate to obtain X-ray crystallographic data for a prototype.

The molecule of the title compound (Fig. 1) shows no unusual geometric features, with the S1—N1 (1.7116 (19) Å) and S1—C1 (1.721 (2) Å) bond distances corresponding to those observed in similar structures (Cavalca *et al.*, 1970; Ranganathan *et al.*, 2002; Steinfeld & Kersting, 2006; Kim *et al.*, 1996; Xu *et al.*, 2006; Sarma & Mugesh, 2007). The N1—N2 bond distance (1.364 (2) Å) is just significantly shorter than that observed in 4,5-dimethyl-2-(3-nitrobenzenesulfonylamino)isothiazol-3(2*H*)-one 1,1-dioxide (1.387 (4) Å; Kolberg *et al.*, 1999). The benzoisothiazole rings system is essentially planar (maximum deviation 0.019 (4) Å for atom C4) and forms a dihedral angle of 67.37 (6)° with the plane of the C8–C13 benzene ring. In the crystal structure (Fig. 2), molecules are linked into chains running parallel to the *b* axis by intermolecular N—H···O and C—H···O hydrogen bonding interactions (Table 1).

### Experimental

The title compound was synthesized as described elsewhere (Vicini *et al.*, 2009). Freshly prepared chlorocarbonylsulfenylchloride (18 mmol) in dried CCl_4_ (40 ml) was added dropwise to a stirred, ice-cooled solution of 2-tosylhydrazine (20 mmol) in pyridine (18 ml). After 2 h the reaction mixture was allowed to cool to room temperature and the crude product was filtered, purified by base-acid (Na_2_CO_3_—HCl) exchange and silica-gel column chromatography (eluent CH_2_Cl_2_—EtOH 95:5 *v*/*v*). Pale yellow single crystals of the title compound suitable for X-ray analysis were obtained by slow evaporation of an ethanol solution at room temperature.

### Refinement

The H atoms bound to the N2 atom was located in a difference Fourier map and refined isotropically with the N—H distance constrained to 0.87 (1) Å. All other H atoms were placed at calculated positions and refined using a riding model, with C—H = 0.93–0.96 Å, and with *U*_iso_(H) = 1.2 *U*_eq_(C) or 1.5 *U*_eq_(C) for methyl H atoms.

### Crystal data

**Table:**

C_14_H_12_N_2_O_3_S_2_	*F*(000) = 664
*M**_r_* = 320.38	*D*_x_ = 1.460 Mg m^−^^3^
Monoclinic, *P*2_1_/*n*	Mo *K*α radiation, λ = 0.71073 Å
Hall symbol: -P 2yn	Cell parameters from 1208 reflections
*a* = 8.051 (3) Å	θ = 3.1–54.7°
*b* = 7.655 (3) Å	µ = 0.38 mm^−^^1^
*c* = 23.91 (1) Å	*T* = 296 K
β = 98.490 (8)°	Prism, pale yellow
*V* = 1457.4 (10) Å^3^	0.28 × 0.26 × 0.12 mm
*Z* = 4	

### Data collection

**Table:**

Bruker SMART 1000 CCD area-detector diffractometer	3521 independent reflections
Radiation source: fine-focus sealed tube	1888 reflections with *I* > 2σ(*I*)
graphite	*R*_int_ = 0.041
ω scans	θ_max_ = 28.0°, θ_min_ = 1.7°
Absorption correction: multi-scan (*SADABS*; Bruker, 1997)	*h* = −10→10
*T*_min_ = 0.892, *T*_max_ = 0.957	*k* = −10→10
17685 measured reflections	*l* = −31→31

### Refinement

**Table:**

Refinement on *F*^2^	Primary atom site location: structure-invariant direct methods
Least-squares matrix: full	Secondary atom site location: difference Fourier map
*R*[*F*^2^ > 2σ(*F*^2^)] = 0.040	Hydrogen site location: inferred from neighbouring sites
*wR*(*F*^2^) = 0.089	H atoms treated by a mixture of independent and constrained refinement
*S* = 1.00	*w* = 1/[σ^2^(*F*_o_^2^) + (0.0354*P*)^2^] where *P* = (*F*_o_^2^ + 2*F*_c_^2^)/3
3521 reflections	(Δ/σ)_max_ < 0.001
194 parameters	Δρ_max_ = 0.23 e Å^−^^3^
1 restraint	Δρ_min_ = −0.28 e Å^−^^3^

### Special details

**Table:**

Refinement. Refinement of *F*^2^ against ALL reflections. The weighted *R*-factor *wR* and goodness of fit *S* are based on *F*^2^, conventional *R*-factors *R* are based on *F*, with *F* set to zero for negative *F*^2^. The threshold expression of *F*^2^ > σ(*F*^2^) is used only for calculating *R*-factors(gt) *etc*. and is not relevant to the choice of reflections for refinement. *R*-factors based on *F*^2^ are statistically about twice as large as those based on *F*, and *R*- factors based on ALL data will be even larger.

### Fractional atomic coordinates and isotropic or equivalent isotropic displacement parameters (Å^2^)

**Table:**

	*x*	*y*	*z*	*U*_iso_*/*U*_eq_	
S1	0.07092 (8)	0.54275 (7)	0.15129 (2)	0.0674 (2)	
S2	−0.38794 (7)	0.64261 (7)	0.12617 (2)	0.06196 (18)	
O1	−0.25352 (19)	0.2912 (2)	0.21942 (6)	0.0740 (4)	
O2	−0.4086 (2)	0.48129 (18)	0.09686 (6)	0.0795 (5)	
O3	−0.5189 (2)	0.7130 (2)	0.15313 (7)	0.0826 (5)	
N1	−0.1134 (2)	0.4976 (2)	0.17603 (7)	0.0585 (5)	
N2	−0.2283 (3)	0.6277 (2)	0.17766 (8)	0.0692 (5)	
H2	−0.247 (3)	0.670 (3)	0.2089 (6)	0.085 (8)*	
C1	−0.1300 (3)	0.3352 (2)	0.19864 (8)	0.0524 (5)	
C2	0.0211 (2)	0.2380 (2)	0.19334 (8)	0.0469 (5)	
C3	0.0556 (3)	0.0671 (3)	0.21027 (8)	0.0584 (5)	
H3	−0.0221	0.0028	0.2269	0.070*	
C4	0.2040 (3)	−0.0051 (3)	0.20233 (10)	0.0702 (6)	
H4	0.2268	−0.1211	0.2122	0.084*	
C5	0.3219 (3)	0.0936 (3)	0.17950 (10)	0.0796 (7)	
H5	0.4249	0.0432	0.1758	0.096*	
C6	0.2925 (3)	0.2611 (3)	0.16232 (10)	0.0722 (6)	
H6	0.3724	0.3250	0.1467	0.087*	
C7	0.1378 (3)	0.3337 (2)	0.16900 (8)	0.0527 (5)	
C8	−0.3251 (2)	0.7991 (3)	0.08016 (8)	0.0544 (5)	
C9	−0.3198 (3)	0.7587 (3)	0.02474 (10)	0.0815 (7)	
H9	−0.3454	0.6466	0.0111	0.098*	
C10	−0.2757 (4)	0.8877 (4)	−0.01057 (10)	0.0977 (9)	
H10	−0.2729	0.8606	−0.0483	0.117*	
C11	−0.2362 (3)	1.0529 (4)	0.00758 (12)	0.0789 (7)	
C12	−0.2448 (3)	1.0894 (3)	0.06352 (11)	0.0793 (7)	
H12	−0.2203	1.2017	0.0772	0.095*	
C13	−0.2886 (3)	0.9645 (3)	0.09934 (10)	0.0708 (6)	
H13	−0.2935	0.9922	0.1369	0.085*	
C14	−0.1871 (4)	1.1911 (4)	−0.03204 (12)	0.1226 (12)	
H14A	−0.1661	1.2993	−0.0120	0.184*	
H14B	−0.2766	1.2068	−0.0629	0.184*	
H14C	−0.0874	1.1549	−0.0464	0.184*	

### Atomic displacement parameters (Å^2^)

**Table:**

	*U*^11^	*U*^22^	*U*^33^	*U*^12^	*U*^13^	*U*^23^
S1	0.0837 (4)	0.0494 (3)	0.0742 (4)	−0.0037 (3)	0.0287 (3)	0.0081 (3)
S2	0.0711 (4)	0.0537 (3)	0.0623 (4)	0.0114 (3)	0.0140 (3)	0.0072 (3)
O1	0.0561 (9)	0.0904 (11)	0.0790 (10)	0.0106 (8)	0.0222 (8)	0.0324 (9)
O2	0.1055 (13)	0.0531 (9)	0.0784 (11)	−0.0047 (8)	0.0092 (9)	−0.0016 (8)
O3	0.0789 (11)	0.0863 (11)	0.0896 (12)	0.0260 (9)	0.0360 (9)	0.0181 (9)
N1	0.0668 (12)	0.0489 (10)	0.0626 (11)	0.0159 (9)	0.0193 (9)	0.0127 (8)
N2	0.0929 (14)	0.0660 (12)	0.0483 (11)	0.0358 (11)	0.0092 (10)	0.0007 (10)
C1	0.0538 (13)	0.0556 (13)	0.0484 (12)	0.0027 (10)	0.0099 (10)	0.0099 (10)
C2	0.0457 (12)	0.0437 (11)	0.0508 (11)	0.0001 (9)	0.0055 (9)	0.0021 (9)
C3	0.0598 (14)	0.0514 (13)	0.0610 (13)	0.0000 (10)	−0.0004 (11)	0.0053 (10)
C4	0.0759 (17)	0.0548 (13)	0.0760 (16)	0.0139 (13)	−0.0013 (13)	−0.0038 (12)
C5	0.0670 (16)	0.0808 (18)	0.0911 (18)	0.0205 (14)	0.0121 (14)	−0.0180 (14)
C6	0.0622 (15)	0.0765 (16)	0.0834 (16)	−0.0070 (13)	0.0287 (13)	−0.0146 (14)
C7	0.0552 (13)	0.0498 (12)	0.0543 (12)	−0.0030 (10)	0.0122 (10)	−0.0045 (9)
C8	0.0579 (13)	0.0556 (12)	0.0491 (12)	0.0104 (10)	0.0056 (10)	0.0034 (10)
C9	0.111 (2)	0.0751 (16)	0.0603 (16)	−0.0120 (15)	0.0195 (14)	−0.0075 (13)
C10	0.125 (2)	0.119 (2)	0.0504 (15)	−0.0115 (19)	0.0165 (15)	0.0051 (16)
C11	0.0669 (16)	0.0886 (19)	0.0774 (19)	−0.0017 (14)	−0.0021 (13)	0.0278 (16)
C12	0.0940 (19)	0.0613 (15)	0.0808 (19)	−0.0026 (13)	0.0065 (14)	0.0065 (14)
C13	0.0920 (18)	0.0637 (15)	0.0571 (14)	0.0010 (13)	0.0125 (13)	−0.0009 (12)
C14	0.108 (2)	0.143 (3)	0.114 (2)	−0.014 (2)	0.0065 (18)	0.070 (2)

### Geometric parameters (Å, °)

**Table:**

S1—N1	1.7116 (19)	C5—H5	0.9300
S1—C7	1.721 (2)	C6—C7	1.394 (3)
S2—O2	1.4175 (16)	C6—H6	0.9300
S2—O3	1.4205 (15)	C8—C13	1.364 (3)
S2—N2	1.647 (2)	C8—C9	1.368 (3)
S2—C8	1.751 (2)	C9—C10	1.380 (3)
O1—C1	1.223 (2)	C9—H9	0.9300
N1—N2	1.364 (2)	C10—C11	1.359 (3)
N1—C1	1.370 (2)	C10—H10	0.9300
N2—H2	0.844 (9)	C11—C12	1.378 (3)
C1—C2	1.448 (3)	C11—C14	1.511 (3)
C2—C3	1.385 (3)	C12—C13	1.364 (3)
C2—C7	1.386 (2)	C12—H12	0.9300
C3—C4	1.355 (3)	C13—H13	0.9300
C3—H3	0.9300	C14—H14A	0.9600
C4—C5	1.387 (3)	C14—H14B	0.9600
C4—H4	0.9300	C14—H14C	0.9600
C5—C6	1.356 (3)		
			
N1—S1—C7	89.03 (9)	C5—C6—H6	121.3
O2—S2—O3	120.93 (11)	C7—C6—H6	121.3
O2—S2—N2	109.30 (10)	C2—C7—C6	120.65 (19)
O3—S2—N2	103.69 (10)	C2—C7—S1	112.83 (15)
O2—S2—C8	107.99 (10)	C6—C7—S1	126.50 (17)
O3—S2—C8	109.17 (10)	C13—C8—C9	120.0 (2)
N2—S2—C8	104.56 (10)	C13—C8—S2	119.47 (17)
N2—N1—C1	123.02 (17)	C9—C8—S2	120.50 (18)
N2—N1—S1	119.29 (14)	C8—C9—C10	118.6 (2)
C1—N1—S1	117.36 (13)	C8—C9—H9	120.7
N1—N2—S2	119.16 (15)	C10—C9—H9	120.7
N1—N2—H2	120.6 (16)	C11—C10—C9	122.7 (2)
S2—N2—H2	114.6 (16)	C11—C10—H10	118.7
O1—C1—N1	122.89 (18)	C9—C10—H10	118.7
O1—C1—C2	129.65 (18)	C10—C11—C12	117.1 (2)
N1—C1—C2	107.45 (17)	C10—C11—C14	121.4 (3)
C3—C2—C7	120.20 (18)	C12—C11—C14	121.5 (3)
C3—C2—C1	126.50 (18)	C13—C12—C11	121.5 (2)
C7—C2—C1	113.30 (17)	C13—C12—H12	119.3
C4—C3—C2	119.2 (2)	C11—C12—H12	119.3
C4—C3—H3	120.4	C12—C13—C8	120.2 (2)
C2—C3—H3	120.4	C12—C13—H13	119.9
C3—C4—C5	120.1 (2)	C8—C13—H13	119.9
C3—C4—H4	119.9	C11—C14—H14A	109.5
C5—C4—H4	119.9	C11—C14—H14B	109.5
C6—C5—C4	122.3 (2)	H14A—C14—H14B	109.5
C6—C5—H5	118.8	C11—C14—H14C	109.5
C4—C5—H5	118.8	H14A—C14—H14C	109.5
C5—C6—C7	117.5 (2)	H14B—C14—H14C	109.5

### Hydrogen-bond geometry (Å, °)

**Table:**

*D*—H···*A*	*D*—H	H···*A*	*D*···*A*	*D*—H···*A*
N2—H2···O1^i^	0.85 (2)	1.95 (2)	2.784 (3)	168 (2)
C6—H6···O2^ii^	0.93	2.56	3.492 (3)	175

Symmetry codes: (i) −*x*−1/2, *y*+1/2, −*z*+1/2; (ii) *x*+1, *y*, *z*.

## References

[bb1] Altomare, A., Burla, M. C., Camalli, M., Cascarano, G. L., Giacovazzo, C., Guagliardi, A., Moliterni, A. G. G., Polidori, G. & Spagna, R. (1999). *J. Appl. Cryst.* **32**, 115–119.

[bb2] Bruker (1997). *SMART*, *SAINT* and *SADABS* Bruker AXS Inc., Madison, Wisconsin, USA.

[bb3] Cavalca, L., Gaetani, A., Mangia, A. & Pelizzi, G. (1970). *Gazz. Chim. Ital.* **100**, 629–638.

[bb4] Clerici, F., Gelmi, M. L., Pellegrino, S. & Pocar, D. (2007). *Top. Heterocycl. Chem.* **9**, 179–264.

[bb5] Farrugia, L. J. (1997). *J. Appl. Cryst.* **30**, 565.

[bb6] Keller, E. (1997). *SCHAKAL97* University of Freiburg, Germany.

[bb7] Kim, W., Dannaldson, J. & Gates, K. S. (1996). *Tetrahedron Lett.* **37**, 5337–5340.

[bb8] Kolberg, A., Sieler, J. & Schulze, B. (1999). *J. Heterocycl. Chem.* **36**, 1081–1086.

[bb9] Nardelli, M. (1995). *J. Appl. Cryst.* **28**, 659.

[bb10] Ranganathan, S., Muraleedharan, K. M., Bharadwaj, P., Chatterji, D. & Karle, I. (2002). *Tetrahedron*, **58**, 2861–2874.

[bb11] Sarma, B. K. & Mugesh, G. (2007). *J. Am. Chem. Soc.* **129**, 8872–8881. 10.1021/ja070410o 17585764

[bb12] Sheldrick, G. M. (2008). *Acta Cryst.* A**64**, 112–122. 10.1107/S0108767307043930 18156677

[bb13] Siegemund, A., Taubert, K. & Schulze, B. (2002). *Sulfur Rep.* **23**, 279-319.

[bb14] Steinfeld, G. & Kersting, B. (2006). *Z. Anorg. Allg. Chem.* **632**, 2010–2016.

[bb15] Vicini, P., Amoretti, L., Tognolini, M., Ballabeni, V. & Barocelli, E. (2000). *Bioorg. Med. Chem.* **8**, 2355–2358. 10.1016/s0968-0896(00)00168-1 11026548

[bb16] Vicini, P., Incerti, M., La Colla, P., Collu, G., Pezzullo, M., Giliberti, G. & Loddo, R. (2009). *J. Med. Chem.* Submitted.

[bb17] Vicini, P., Manotti, C., Caretta, A. & Amoretti, L. (1997). *Arzneim. Forsch. Drug Res.* **47**, 1218–1221. 9428977

[bb18] Vicini, P., Zani, F., Cozzini, P. & Doytchinova, I. (2002). *Eur. J. Med. Chem.* **37**, 553–564. 10.1016/s0223-5234(02)01378-8 12126774

[bb19] Xu, F.-L., Lin, Q. & Yin, X.-Q. (2006). *Acta Cryst.* E**62**, o496–o497.

[bb20] Zani, F., Vicini, P. & Incerti, M. (2004). *Eur. J. Med. Chem.* **39**, 135–140. 10.1016/j.ejmech.2003.11.004 14987822

